# Identification of IL-28B Genotype Modification in Hepatocytes after Living Donor Liver Transplantation by Laser Capture Microdissection and Pyrosequencing Analysis

**DOI:** 10.1155/2018/1826140

**Published:** 2018-03-04

**Authors:** King-Wah Chiu, Toshiaki Nakano, Kuang-Den Chen, Tsung-Hui Hu, Chih-Che Lin, Li-Wen Hsu, Chao-Long Chen, Shigeru Goto

**Affiliations:** ^1^Division of Hepato-Gastroenterology, Department of Internal Medicine, Kaohsiung Chang Gung Memorial Hospital, Kaohsiung, Taiwan; ^2^College of Medicine, Chang Gung University, Taoyuan, Taiwan; ^3^Liver Transplantation Center, Kaohsiung Chang Gung Memorial Hospital, Kaohsiung, Taiwan; ^4^Graduate Institute of Clinical Medical Sciences, Chang Gung University, Taoyuan, Taiwan; ^5^Institute for Translational Research in Biomedicine, Kaohsiung Chang Gung Memorial Hospital, Kaohsiung, Taiwan; ^6^Division of General Surgery, Department of Surgery, Kaohsiung Chang Gung Memorial Hospital, Kaohsiung, Taiwan; ^7^Fukuoka Institution of Occupational Health, Nobeoka, Japan; ^8^Department of Nursing, Josai International University, Togane, Japan

## Abstract

The aim of this study is to elucidate the biogenetic modification of donor and recipient interleukin-28B (IL-28B) genotypes in liver graft biopsies after living donor liver transplantation (LDLT) for chronic hepatitis C virus- (HCV-) related, end-stage liver disease. Fifty liver graft biopsies were collected from recipients during LDLT treatment for HCV-related, end-stage liver disease. DNA was extracted from all 50 liver tissues, and the IL-28B single-nucleotide polymorphisms (SNPs) rs8099917 and rs12979860 were studied for allelic discrimination by real-time PCR analysis. Blood samples were obtained from donors and recipients on postoperative day 0 (POD0), POD7, and POD30. We randomly selected five liver biopsies and isolated the hepatocytes by laser capture microdissection (LCM) to evaluate genotype modifications resulting from LDLT. After LDLT, the IL-28B SNP rs8099917 was identified not only in the liver graft biopsies and donors' sera (TT = 41 : 43; GT = 9 : 5; GG = 0 : 2), but also in liver graft biopsies and recipients' sera on POD0 (TT = 41 : 44; GT = 9 : 4; GG = 0 : 2), POD7 (TT = 41 : 30; GT = 9 : 18; GG = 0 : 2), and POD30 (TT = 41 : 29; GT = 9 : 19; GG = 0 : 2). A significant difference was observed between the rs8099917 allele frequencies of liver graft biopsies and recipients' sera on POD30 (*p* = 0.039). In addition, a significant difference was also noted between the rs12979860 allele frequencies of liver graft biopsies and donors' sera (CT = 49 : 39; TT = 1 : 10) (*p* = 0.012) and of liver graft biopsies and recipients' sera on POD0 (CT = 49 : 39; TT = 1 : 11) (*p* = 0.002), POD7 (CT = 49 : 42; TT = 1 : 8) (*p* = 0.016), and POD30 (CT = 49 : 41; TT = 1 : 9) (*p* = 0.008). This phenomenon was confirmed by pyrosequencing of hepatocytes isolated by LCM. Following LDLT, the TT-to-GT IL-28B genotype modification predominated in rs8099917, and the CC-to-CT modification predominated in rs12979860. In conclusion, these modified phenomena suggested that the selected donor with a predictable and favourable IL-28B genotype will not confer a benefit on the recipient in the living donor liver transplantation setting.

## 1. Introduction

In hepatitis C virus (HCV) infection control, human interleukin 28B (IL-28B) genetics may play a role in patients' selection and treatment decisions. The most common IL-28B single-nucleotide polymorphisms (SNPs) are rs8099917 and rs12979860, which are associated with spontaneous clearance or sustained viral response with interferon treatment of HCV genotype 1 [1, 2]. Patients with the favourable rs12979860 CC genotype show better treatment responses, particularly among Asians [3, 4]. IL-28B originates from an individual's own chromosome. Since the liver is the primary target organ and replication site of HCV, possible modification or dominant expression of IL-28B genotype has been confirmed in liver grafts after living donor liver transplantation (LDLT). However, a discrepancy in IL-28B genotypes between recipients and donor grafts and the related efficacy in preventing HCV recurrence after LDLT remain unelucidated. Therefore, it is worth studying the effect of different IL-28B genotypes on HCV replication between donors and recipients after LDLT. The dominant IL-28B genotype expressed in the peripheral blood of recipients after LDLT may be derived from either the donor or the recipient. However, the possibility that a stem cell component may be transferred with the liver graft is not discarded. Herein, we investigated the biogenetic modification of IL-28B SNPs rs8099917 and rs12979860 in liver graft biopsies and sera after LDLT. Hepatocytes were isolated from the liver biopsy by laser capture microdissection (LCM), and IL-28B modification was explored by pyrosequencing analysis. The current study would like to identify the evidence of the modification on IL-28B rs8099917 and rs12979860 in different genotypes of donor and recipients undergo LDLT.

## 2. Methods

### 2.1. Ethics Statement

This research was conducted in accordance with the World Medical Association Declaration of Helsinki (2000) and our own institutional standards and was approved by the Institutional Review Board of Chang Gung Memorial Hospital (Approval number 103-0679C). Written informed consent for participation in this study was obtained from each participant or from a parent or guardian for child participants.

### 2.2. Liver Biopsy Tissue Specimens

Fifty liver graft biopsies were obtained from recipients after LDLT when elevated liver function was observed or acute rejection was suspected clinically, as described [5, 6]. DNA was extracted from all liver tissues, and IL-28B SNP rs8099917 and rs12979860 allele frequencies were studied by real-time polymerase chain reaction (PCR) analysis. All samples were obtained from the Liver Transplantation Centre of Kaohsiung Chang Gung Memorial Hospital.

### 2.3. Peripheral Blood Serum Samples

We studied samples from 50 donor and recipient pairs who underwent LDLT in our Liver Transplantation Centre. Blood samples were obtained from the donors and recipients at three different time points: postoperative day 0 (POD0), which represented the initial recipients' status mixed with donors' characteristics; POD7, which represented an adjustment period with active immune responses between liver grafts and peripheral mononuclear cells; and POD30, which represented a late, stabilised status after LDLT. The clinical profiles of the 50 pairs of donor and recipient were showed on Table 1.

### 2.4. IL-28B Genotypes

IL-28B SNP rs8099917 and rs12979860 genotypes were studied in liver tissues, donor's blood samples, and recipient's blood samples on POD0, POD7, and POD30, using a 7500 Fast Real-Time PCR System (Applied Biosystems, Foster City, CA, USA) with Custom TaqMan SNP Genotyping Assays (Applied Biosystems) for allele discrimination.

### 2.5. LCM Experiments

Liver graft biopsy tissues were stored at −80°C in OCT compound, and unstained 5 *μ*m single-layer frozen sections were subjected to LCM, using a laser microdissection and pressure-catapulting microscopic system (LMPC, Carl Zeiss, München, Germany). Over 4,000 single-layer cells were pooled per sample, dissolved in lysis buffer, and DNA was extracted after PCR amplification.

### 2.6. Pyrosequencing Analysis

Genomic DNA was extracted from liver graft tissues and peripheral blood using a genomic DNA purification kit followed by biotinylated DNA PCR amplification. The rs8099917 SNP was identified by PCR-restriction fragment length polymorphism (PCR-RFLP) assay. Genotyping of the rs8099917 SNP was performed by pyrosequencing with a PyroMark Q24 System (Qiagen, Hilden, Germany), according to the manufacturer's recommended protocol. Primers were designed for pyrosequencing using PyroMark Assay Design Software, version 2.0. For the PyroMark PCR assay, we used a forward primer (5′-TGTACCACCCAGCTTAACGA-3′) and a 5′-biotinylated reverse primer (5′-biotin-GGTCCAAACAGGGAAGAAAT-3′) [7]. The assay was performed in a 25 *μ*L reaction volume. The PCR conditions consisted of initial denaturation at 95°C for 15 min, followed by 45 cycles of denaturation at 94°C for 30 s, annealing at 60°C for 30 s, and extension at 72°C for 30 s, with a final extension step at 72°C for 10 min. The PCR products were separated on a 2% agarose gel. The obtained product had a length of 125 bp.

### 2.7. Statistical Analysis

Statistical analyses were performed using SPSS software (version 17.0; SPSS, Chicago, IL, USA). Since the donor sera and recipient liver grafts could be from the same original source, comparisons of the IL-28B rs8099917 and rs12979860 SNP genotypes between the donors and recipients were performed at different time points by the McNemar–Bowker test. For the three haplotypes in the case of rs8099917, 3-by-2 analysis with the TT, GT, and GG genotypes was performed to compare the differences between the recipient liver grafts and donor sera on POD0, POD7, and POD30. In the case of rs12979860, as there would only be one genotype (TT) in the donor serum, a 2-by-2 analysis was performed for CT and CC to evaluate the statistical significance. All liver graft biopsies and recipient serum samples expressed only two genotypes in this study on POD0, POD7, and POD30. *p* values < 0.05 were considered statistically significant.

## 3. Results

### 3.1. Pathological Analysis of Liver Biopsies

Of the 50 recipients, liver biopsies for 18 cases (36%) were performed within 1 month and for 32 cases (64%) were performed more than 1 month after LDLT transplantation. The acute rejection rate was 14% (7/50) in this study. Following tissue biopsy, we found that 54% (27/50) of the allografts presented with chronic hepatitis related to recurrent HCV infection with Ishak's system- (ISHAK-) modified Histology Activity Index (HAI) necroinflammatory scores of 4 to 8 and ISHAK-modified fibrosis scores of 1 to 3. We observed a change in fatty acid contents in 10% (5/50) of the samples, three of which (60%, 3/5) showed a nonalcoholic fatty liver disease- (NAFLD-) activity score of 5, which is diagnostic of nonalcoholic steatohepatitis. We also found that 8% (4/50) and 14% (7/50) of the samples showed cholangitis and nonspecific reactive changes, respectively. In our series, detection of serum HCV-RNA was negative in 12 recipients (24%, 12/50) before LDLT and in 40 recipients (80%, 40/50) after LDLT (*p* < 0.05). The serum HCV-RNA was obtained after the liver biopsy. Antiviral treatment was scheduled based on the parameters of our liver transplantation program, as previously reported.

### 3.2. Modification of IL-28B SNP rs8099917 and rs12979860 Genotypes after LDLT

**Figure 1:**
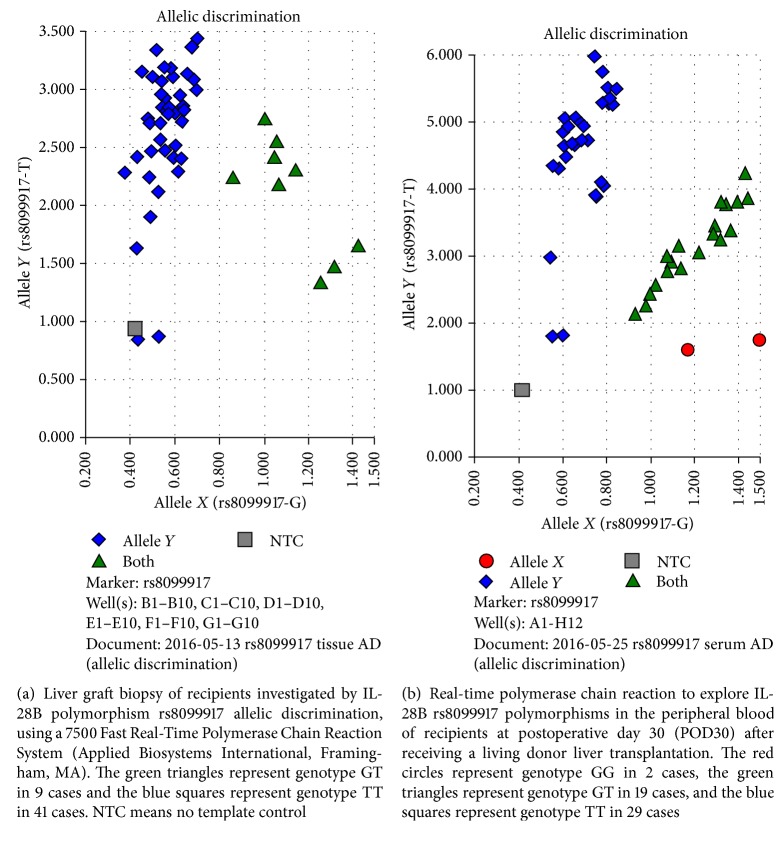


**Figure 2:**
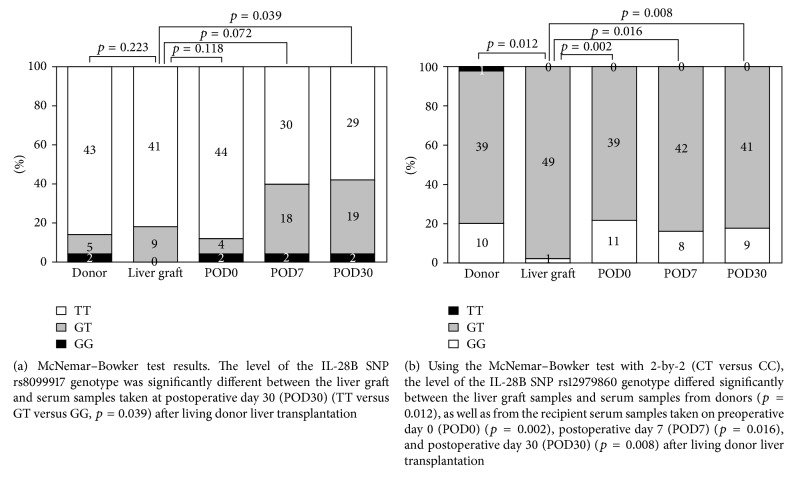


After LDLT, IL-28B rs8099917 genotype modification was identified between the liver graft biopsy tissues (Figure 1(a)) and donors' sera (TT = 41 : 43; GT = 9 : 5; GG = 0 : 2) and between the liver graft biopsy tissues and recipients' sera on POD0 (TT = 41 : 44; GT = 9 : 4; GG = 0 : 2), POD7 (TT = 41 : 30; GT = 9 : 18; GG = 0 : 2), and POD30 (TT = 41 : 29; GT = 9 : 19; GG = 0 : 2) (Figure 1(b)). A significant difference was observed between genotype frequencies in the liver grafts and recipients' sera on POD30 (*p* = 0.039) (Figure 2(a)). In addition, rs12979860 genotype modification differed significantly between liver graft biopsy tissues and donors' sera (CT = 49 : 39; TT = 1 : 10) (*p* = 0.012) and between liver graft biopsies and recipients' sera on POD0 (CT = 49 : 39; TT = 1 : 11) (*p* = 0.002), POD7 (CT = 49 : 42; TT = 1 : 8) (*p* = 0.016), and POD30 (CT = 49 : 41; TT = 1 : 9) (*p* = 0.008) (Figure 2(b)).

### 3.3. Pyrosequencing Analysis of IL-28B rs8099917 Used to Compare Isolated Hepatocytes with Liver Graft Biopsies and Recipient Serum Samples

**Figure 3:**
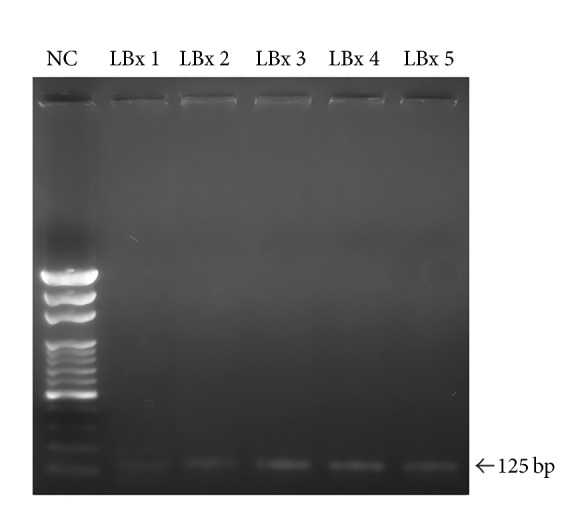
Interleukin-28B single-nucleotide polymorphism DNA was identified using a polymerase chain reaction-restriction fragment length polymorphism (PCR-RFLP) assay with a 125-base pair band. NC, normal control; LBx, liver biopsy.

**Figure 4:**
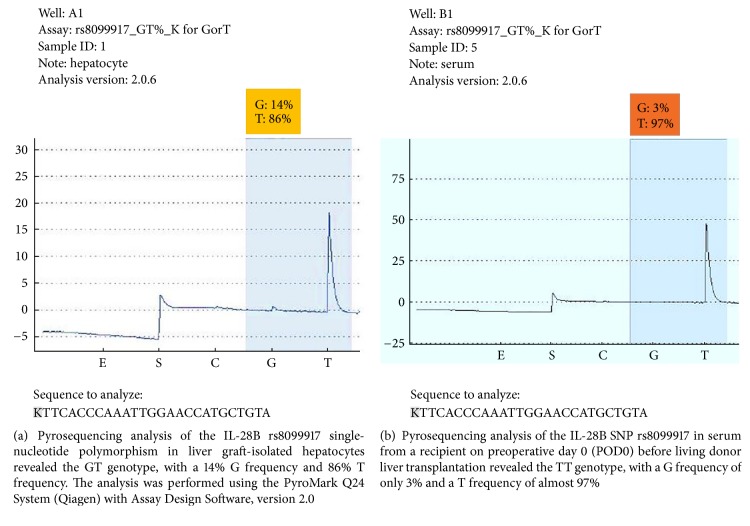


Based on the donors' serum IL-28B rs8099917 genotypes, five cases (including genotype GG in one case, GT in two cases, and TT in two cases) served as index cases for choosing related liver graft biopsies when clinically required after LDLT. We analyzed the liver tissues from the five recipients to isolate the hepatocytes via automated and noncontact LCM. Single-cell LCM was performed to capture over 4,000 hepatocytes in grids and preserve their genomic relationships. This process was followed by pyrosequencing. For each of the five randomised cases, the isolated hepatocytes from liver graft biopsy tissue and a given recipient's serum on POD0 were analyzed by PCR-RFLP (Figure 3) and subsequent pyrosequencing (Table 2). The original IL-28B rs8099917 SNP genotype of the donor in one case was GG. After LDLT, however, the genotype of the graft hepatocytes mutated to GT with 14.45% G and 85.55% T allelic frequencies (Figure 4(a)), whereas the genotype of the recipient's serum on POD0 was TT with 3.36% G and 96.64% T allelic frequencies (Figure 4(b)). With respect to the two donors originally harbouring the TT genotype, the genotypes of the graft hepatocytes remained unchanged, as they exhibited G/T ratios of 5.98%/94.02% and 2.7%/97.3% at each time point, respectively. The genotypes of recipient serum samples on POD0 were also TT, with G/T ratios of 2.34%/97.66% and 2.07%/97.93%, respectively. The original genotype of the fourth donor was GT, as was the genotype of the corresponding isolated hepatocytes with 16.54% G and 83.46% T allelic frequencies. However, the genotype of the recipient's serum on POD0 was TT with 3.15% G and 96.85% T allelic frequencies. Finally, for the fifth donor, the genotype of both the isolated hepatocytes and the recipient's serum on POD0 was GT, with G/T ratios of 86.43%/33.57% and 59.02%/40.98%, respectively.

## 4. Discussion

Although IL-28B is encoded in the human genome (19q), in this study, we found that some genotypes were time-dependently modified after LDLT from TT (in a given donor's serum) to GT (in the corresponding liver graft biopsy tissue) in rs8099917 and from CC to CT in rs12979860. Allelic discrimination becomes even more complex when sera following LDLT are examined. Moreover, leukocyte and stem cell populations from the donor's liver will in effect contaminate the blood samples. For example, a recipient's TT genotype with a donor's GG liver genotype may be identified as a GT heterozygote, as a fraction of the peripheral blood mononucleocyte population from the donor's liver is present in the blood sample. Although the ratio of host TT cells to donor GG cells may be high, when quantified by qPCR, it may still be observed as a heterozygote, even though two distinct cell populations are present. The same applies when genotyping graft tissue that may be infiltrated with numerous host immune cells with a different genotype; hence explaining the differences we found in the donors' sera and liver graft tissue genotypes. LCM has been previously shown to be a useful method for purifying the original source when isolating individual hepatocytes to explore the IL-28B rs8099917 genotype [8, 9]. As demonstrated in our previous study on CYP2C19 genotypes, the characteristics of a donor's genotype do not affect the expression of the recipient's genes after LDLT [10]. This phenomenon could be unstable at an early stage depending on the immune reaction and whether there is acute rejection at any extent on POD0 through POD7 [5, 6]. Possible modifications and/or the determination of dominant expression will occur after organ transplantation in association with clinical and immunological turbulence or stabilisation after transplantation. Many clinical observations have suggested that donor liver grafts carrying the clinically favourable IL-28B allele may have a beneficial effect in terms of improving sustained viral responses and decreasing the 5-year mortality rate of recipients with HCV infection [11–13]. In fact, immunological responses are extremely complex in the context of liver transplantation if a recipient has two different IL-28B genotypes owing to the contributing sources of the recipient's own genes and the donor allograft. In our study, the genotypes of IL-28B rs8099917 and rs12979860 in the isolated hepatocytes from the liver graft and peripheral blood from the recipient were significantly different. This is an exciting discovery with regard to the treatment responses of recipients with HCV infection after liver transplantation. In our previous study on the cytochrome P450 system, a similar phenomenon was observed with modified SNPs genotypes in CYP2C19, CYP3A4, CYP3A5, and MDR1-3435, when the genotypes of the donor allograft and the recipients were different [14]. Our results suggest that the recipient's liver mutated to a favourable IL-28B genotype such as rs8099917 TT or rs12979860 CC. Further clinical investigation should be conducted to determine whether a recipient's serum expresses different IL-28B alleles to reduce the impact of adverse post-LDLT outcomes based on who (i.e., the recipient or the donor) carries the dominant genotype. Kandathil et al. demonstrated that the HCV was distributed via clustering in hepatocytes [9]. The favourable IL-28B rs12979860 CC genotype in a donor allograft results in better outcomes in terms of spontaneous HCV clearance in the target hepatocytes. Final expression levels of IL-28B alleles in a recipient's serum are impacted by the donor's allograft and the recipient's immune system, which may contribute to the genotype of HCV itself. It may be possible that the difficult-to-treat HCV, such as genotype 1b, may impact the recipient's IL-28B alleles to become an unfavourable genotype after LDLT. In contrast, the favourable IL-28B may be modified by the stem cell after liver transplantation in case of HCV genotype 2 or 3. These conditions could affect the recipient-derived inflammatory infiltrates within the biopsy tissue and thus influence the proportion of IL-28B polymorphisms derived from recipient versus donor. Such variation in allograft pathology needs to be taken into account when interpreting the results of this analysis. Some limitations of the present study must be acknowledged. Owing to budgetary limitations, we did not conduct LCM and pyrosequencing for all 50 LDLT recipients. Even in the five subjects where LCM was carried out, it was not possible to exclude some contamination of excised hepatocyte material by leukocytes, particularly with up to 4,000 cells excised, which may have contributed to the results obtained. Based on the current results, although the IL-28B rs8099917 TT and rs12979860 CC genotypes are associated with improved treatment outcomes when interferon is not used to treat HCV infection, attempting to match the genotype of donor graft may not provide the desired result. After liver transplantation, the IL-28B gene may become modified owing to various reasons, and it cannot be ruled out that different HCV genotypes cause changes in the IL-28B gene after liver transplantation. Based on our liver biopsy results, the effects of HCV are generally identified based on a prior history of chronic infection, even when the serum is negative for HCV-RNA. It represents HCV which may move from the serum into hepatocytes upon immunological responses during suppressive antiviral treatment. Thus, in future studies, it will be important to study HCV genotypes, viral loads before and after liver transplantation, and sustained viral responses (even after HCV in liver-transplanted patients had disappeared from liver cells) to introduce protective antibodies against HCV. As discussed by Sheahan and Rice, such avenues of investigation are no longer science fiction [15]. Finally, these modified phenomena suggested that the selected donor with a predictable and favourable IL-28B genotype will not confer a benefit on the recipient in the living donor liver transplantation setting. The current study would like to identify the evidence of the modification on IL-28B rs8099917 and rs12979860 in different genotypes of donor and recipients undergo LDLT. In our results, this pattern reflects that the final genotype of IL-28B would not be predictable. At the meanwhile, this modification of IL-28B genotypes does not affect the practical consequences for patients in terms of reinfection or future retreatment or rejection. To treat the virus itself with the current direct acting antiviral agent is the best way on the outcome of HCV-related LDLT.

In conclusion, IL-28B genotype modification was predominant, not only in terms of the TT-to-GT modification in the rs8099917 SNP, but also in terms of the CC-to-CT modification in the rs12979860 SNP. This phenomenon was determined by pyrosequencing after microdissection of isolated hepatocytes following LDLT.

## Figures and Tables

**Table 1:** The clinical profiles of the 50 pairs of donor/recipient in living donor liver transplantation.

Category	Donor	Recipient										*p* value
Age (mean) years	20–54 (34.5)	48–72 (59.14)										0.000
Gender, male/female	26/24	22/28										>0.05
		Before LDLT					After LDLT					
ALT	19.00 ± 6.36	65.30 ± 17.68					23.12 ± 7.98					
AST	17.23 ± 5.78	99.89 ± 34.56					20.17 ± 8.28					
Bilirubin, total	0.64 ± 0.21	1.17 ± 0.42					0.78 ± 0.34					
HCV-RNA												
Positive (%)		44 (88)					26 (52)					0.000
Negative (%)		6 (12)					24 (48)					0.000
Genotype												
G1a		4					2					
G1b		29					19					
G2a		7					2					
G2b		3					1					
G3b		1					1					
Uncertain		6					6					
IL-28B rs8099917												
TT (%)	43 (86.0)	41 (82)					41 (82)					>0.05
GT (%)	4 (8.0)	8 (16)					9 (18)					>0.05
GG (%)	3 (6.0)	1 (2)					0 (0)					>0.05
IL-28B rs12979860												
CC (%)	7 (14.0)	1 (2)					4 (8)					>0.05
CT (%)	43 (86.0)	49 (96)					46 (92)					>0.05
TT (%)	0 (0)	0 (0)					0 (0)					

*Antiviral treatment strategy* ^*∗*^		a	b	c	d	e	a	b	c	d	e	
rs8099917												
TT (%)	22 (91.7)	4	4	4	6	3	4	4	4	6	3	
GT (%)	1 (4.2)		1			2		1			2	
GG (%)	1 (4.2)											
rs12979860												
CC (%)	2 (8.3)				1					1	2	
CT (%)	22 (91.7)	4	5	4	5	5	4	5	4	5	3	
TT (%)	0 (0)											

^*∗*^Only 24 recipients with HCV-RNA clearance after living donor liver transplantation and their antiviral treatment strategy including (a) no treatment; (b) history of pegIFN/RBV; (c) pretransplant pegIFN/RBV; (d) posttransplant pegIFN/RBV; (e) DAA (direct-acting antiviral agent).

**Table 2:** The results of the pyrosequencing investigation of the interleukin 28B single nucleotide polymorphism rs8099917 genotypes on the isolated hepatocytes from the liver graft biopsy and the serum from the recipients on the postoperative day 0 and the relationship of the donor's genotype.

Donor (serum)	Liver graft (cell)			POD0 (serum)		
Genotype	G freq. (%)	T freq. (%)	Genotype	G freq. (%)	T freq. (%)	Genotype
GG	14.45	85.55	GT	3.36	96.64	TT
TT	5.98	94.02	TT	2.34	97.66	TT
TT	2.7	97.3	TT	2.07	97.93	TT
GT	16.54	83.46	GT	3.15	96.85	TT
GT	66.43	33.57	GT	59.02	40.98	GT

Genotype = genomic DNA that is identified by the Applied Biosystems International, 7500 real-time polymerase chain reaction; freq. = frequency (pyrosequencing method, PyroMark, Qiagen).

## Abbreviations

ABI: Applied Biosystems International
HCV: Hepatitis C virus
IL-28B: Interleukin-28B
LCM: Laser capture microdissection
LDLT: Living donor liver transplantation
PCR: Polymerase chain reaction
SNP: Single-nucleotide polymorphism.
